# A neonatal case of vascular ring with Alagille syndrome

**DOI:** 10.1177/2050313X231197321

**Published:** 2023-09-02

**Authors:** Pei-Shan Lee, Jose A Silva Sepulveda, Miguel Del Campo, Sandra L Leibel, Amber Hildreth, Krishelle L Marc-Aurele

**Affiliations:** 1 Department of Pediatrics, UC San Diego, La Jolla, CA, USA; 2 Department of Pediatrics, Rady Children’s Hospital, San Diego, CA, USA

**Keywords:** Vascular ring, Alagille syndrome, cholestasis, cardiovascular anomalies, JAG1

## Abstract

A female infant, born at 37 week 5 days to a mother via induced vaginal delivery for preeclampsia, was prenatally diagnosed with a right aortic arch with vascular ring. On the third day of life, the infant exhibited a bronze-gray coloration, and a direct bilirubin of 1.7 mg/dL was detected. The abdominal ultrasound did not visualize the gallbladder. Clinically, the infant displayed features consistent with Alagille syndrome, including unusual facial appearance, butterfly vertebrae, cardiovascular defects, and cholestasis. The geneticist noted that the mother of the patient also exhibited similar features. Both the infant and the mother were diagnosed with Alagille syndrome, both having the same heterozygous JAG1 gene (NM_000214.2) variant (c.1890_1893del, p.Ile630Metfs*112). We believe that the vascular ring observed in our patient is the first reported instance of a vascular ring associated with Alagille syndrome.

## Introduction

Alagille syndrome (ALGS) is an autosomal dominant syndrome with variable penetrance affecting multiple organs: liver, kidney, heart, blood vessels, skeleton, and eyes. It has a prevalence of 1 in 30,000 live birth.^1^ The majority of patients present prior to 6 months of age, usually due to neonatal cholestasis like this patient and later failure to thrive.^2^ We present a case of a newborn with ALGS that presented with cholestasis and the first-ever documented diagnosis of a vascular ring.

## Case

A 37-week 5-day-old female infant was delivered via induced vaginal delivery for preeclampsia. The pregnancy was complicated by well-controlled gestational diabetes requiring insulin, a history of COVID-19 infection, and the mother being a carrier status for Fragile X. Prenatal diagnosis revealed a right aortic arch with vascular ring in the fetus. Following birth, the infant displayed good vigor and received routine resuscitation, with Apgar scores of 7 and 9 at 1 and 5 min of life, respectively.^3^ Due to the identified prenatal condition, the infant was admitted to the neonatal intensive care unit (NICU) after birth for continuous monitoring.

An echocardiogram performed on the first day of life showed a right aortic arch with aberrant left subclavian artery and left-sided ductus arteriosus, forming a vascular ring and a small ventricular septal defect (VSD). The patent ductus arteriosus closed spontaneously without intervention. Genetic testing for DiGeorge was negative. Additionally, the infant was found to be a premutation carrier for Fragile X syndrome, like the mother.

At 25 h of life, the infant’s total bilirubin was found to be 10.8 mg/dL, and double phototherapy was initiated. The mother stated that she and her siblings all were treated for jaundice as neonates and mentioned a distant relative had hemolytic anemia. However, the infant’s reticulocyte count was not significantly elevated at 5%, and further workup showed an unremarkable complete blood count and testing for glucose-6-phosphate dehydrogenase deficiency was negative. Thyroid-stimulating hormone and free thyroxine were within normal limits. On the third day of life, the infant appeared bronze gray in color and the direct bilirubin was measured at 1.7 mg/dL. Her aspartate aminotransferase (AST), alanine aminotransferase (ALT), and alkaline phosphatase were normal; however, her gamma-glutamyl transferase (GGT) was elevated at 763 U/L. Total bilirubin maximum reached 19 mg/dL at 73 h of life and trended downward appropriately over time.

Pediatric gastroenterology was consulted and recommended further workup. Matrix metalloproteinase-7 was not suggestive of biliary atresia (BA) (58.5 ng/mL, less than 100 ng/mL). Testing for cytomegalovirus was negative and alpha-1 antitrypsin phenotype was MM, indicating a normal result. Abdominal ultrasound revealed two right renal arteries and the gallbladder was not visualized. Additionally, a butterfly vertebra was noted at the T6 level, indicating a skeletal abnormality. A pediatric ophthalmology exam showed bilateral posterior embryotoxon, which is associated with ALGS.

Magnetic resonance angiography (MRA) of the brain was conducted, revealing markedly hypoplastic bilateral internal carotid arteries that did not appear to reach the level of the skull base. The anterior circulation was supplied by the posterior circulation through prominent communicating arteries.

Pediatric genetics was consulted and remarked that the infant’s facial features were consistent with ALGS: broad forehead with triangular face, mild hypertelorism (ICD 2 cm, PFL 1.3 cm), high nasal bridge, bulbous tip of nose, small, pointed chin with narrow mandible, bilateral “railroad track” ears due to prominent curs of helix, bilateral camptodactyly of fifth fingers. Interestingly, the geneticist observed similar features in the patient’s mother. However, the mother had not exhibited any symptoms suggestive of a diagnosis of ALGS.

**Figure 1.**
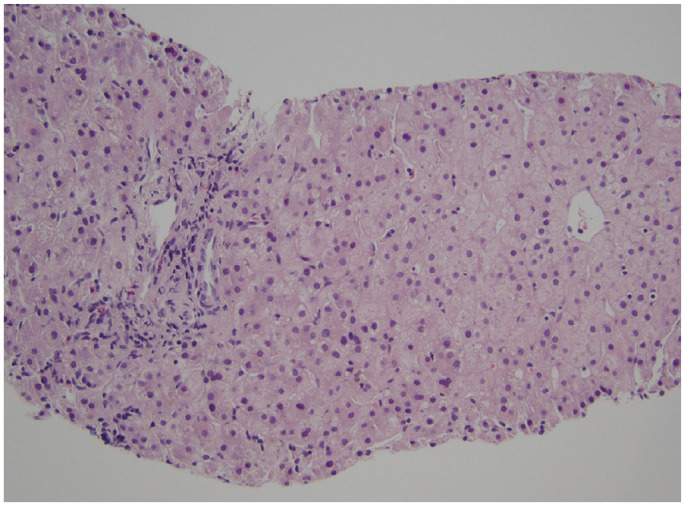
H&E stained section of liver needle biopsy showing portal tract with absent bile duct; overall bile duct to portal tract ratio was 0.4, consistent with paucity of bile ducts/Alagille syndrome. Photo courtesy of Dr. Denise Malicki, Clinical Professor of Pathology at UC San Diego.

A cholestasis genetic panel confirmed the diagnosis of ALGS, which was attributed to a specific heterozygous JAG1 gene mutation (NM_000214.2) variant (c.1890_1893del, p.Ile630Metfs*112). It is much debated whether extrahepatic BA can occur concurrently with ALGS or if it is the Alagille phenotype that may mimic BA. It is worth noting that patients with ALGS who undergo a Kasai procedure have shown poor outcomes.^4^ In response to the parents’ request, a liver biopsy was performed, which revealed a paucity of bile ducts and no histologic evidence of BA (Figure 1). Subsequently, the patient’s mother was also tested, and the results showed the presence of the same JAG1 gene mutation.

Initially, the infant did not have any feeding difficulties or respiratory issues related to her vascular ring. However, after discharge from the NICU, at 10 months of age, she developed signs suggestive of dysphagia with solid foods. In our institution, triple endoscopy (esophagogastroduodenoscopy, laryngoscopy, and bronchoscopy) is performed in all patients with a vascular ring regardless of symptoms to guide decision-making regarding the need for surgery. Her triple endoscopy is scheduled but not yet performed. In most cases, surgical intervention is warranted for symptomatic patients with vascular rings. However, by 12 months of age, she also has shown progression of her liver disease, with a peak total bilirubin of 34 mg/dL, worsening pruritus, and development of portal hypertension prompting listing for liver transplantation.

## Discussion

Cardiovascular anomalies occur in more than 90% of ALGS patients.^5^ In this case, we present the first published instance of a vascular ring anomaly associated with ALGS. Among the various cardiovascular defects seen in ALGS, the most common complex congenital heart defect is Tetralogy of Fallot, which accounts for 7%–12% of cases, while peripheral pulmonary stenosis is the most frequent simple defect, affecting at least 75% of cases.^6^ In a large study, up to 22% of patients had right-sided congenital heart defects (i.e., Tetralogy of Fallot, pulmonary valve stenosis or atresia, etc.), 11% with left-sided congenital heart disease (i.e., coarctation, aortic valve stenosis, etc.), and 15% with atrial septal defect or VSD.^7^ Our patient had a VSD and a right aortic arch. There have also been other reports of right aortic arch anomalies, but we were unable to find other cases of a vascular ring.^7^

It has been described that 10% of Alagille patients have additional blood vessel anomalies including stenoses, which may involve intracranial vessels, the aorta, and renal, celiac, superior mesenteric, and subclavian arteries.^7^ Previous cases of ALGS have also described abnormalities involving the subclavian artery, such as aberrant or hypoplastic subclavian artery.^8,9^ Our patient’s hypoplastic internal carotid arteries, duplicate right renal arteries, aberrant left subclavian artery, and left-sided ductus arteriosus forming a vascular ring all represent regional vessel involvement associated with ALGS.

Interestingly, the baby’s diagnosis led to the mother’s diagnosis of ALGS. Around 50%–70% Alagille patients have a de novo mutation and 30%–50% have an inherited mutation.^10^ There are also reports of parental somatic/germline mosaicism.^10^ More than 40% of inherited JAG1 gene mutations are found after the diagnosis of Alagille in other family members.^6^ For this case, the mother’s presentation reflects Alagille’s variable penetrance and expression: she had characteristic facial features, a history of jaundice at birth, but no other significant clinical history. Liver involvement is present in 80%–100% of patients with ALGS,^1^ but the severity is highly variable including bile duct paucity, conjugated hyperbilirubinemia, chronic cholestasis characterized by pruritus, xanthomas and fat-soluble vitamin deficiencies, and end-stage liver disease. While liver disease with high GGT cholestasis remains the most common presentation of ALGS, not all patients have liver disease.^1^ Considering the mother’s presentation and the identification of the JAG1 gene variant, she meets the revised criteria for the diagnosis of ALGS, which includes a family history of the condition and the presence of a known pathogenic variant in the JAG1 gene.^11^ Recommendations were given to the mother to undergo liver function testing, and an echocardiogram to rule out any associated congenital heart defects.

Vascular rings are considered rare malformations, represent around 1% of congenital heart defects, and can cause patients to be asymptomatic or have symptomatic respiratory and/or gastrointestinal symptoms due to compression of the trachea, esophagus, or both.^12^ Since the introduction of better imaging modalities, prenatal detection has improved with 73.6% of vascular rings diagnosed in utero.^13^ A recent retrospective case review by Peacock et al. reported a high rate of genetic diseases associated with vascular ring, specifically with 22q11.2 mutation being the most common. ALGS was not among the genetic syndromes reported. Surgical intervention is warranted in symptomatic patients and patients typically recover without ongoing symptoms in 93.2% of cases.^13^

## Conclusion

We believe our patient’s vascular ring is the first reported vascular ring associated with ALGS. Vascular ring should be included under the list of less frequent cardiovascular anomalies for ALGS.
